# Chlamydial Pneumonitis: A Creepy Neonatal Disease

**DOI:** 10.1155/2013/549649

**Published:** 2013-02-12

**Authors:** Kam Lun Hon, Alexander K. C. Leung

**Affiliations:** ^1^Prince of Wales Hospital, The Chinese University of Hong Kong, Shatin, Hong Kong; ^2^Alberta Children's Hospital, The University of Calgary, Calgary, AB, Canada T2N 1N4

## Abstract

We present a case of neonatal chlamydial pneumonitis to illustrate that a high index of suspicion is necessary to make the diagnosis so that treatment can be promptly instituted. The child was afebrile and the only symptom was a cough. The respiratory equations are calculated to understand the respiratory physiology. There was no overt abnormality with ventilation, oxygenation, compliance, resistance, or ventilation-perfusion mismatch despite radiographic abnormality. The literature is searched to review if treatment with a systemic macrolide antibiotic is needed in an otherwise asymptomatic neonate with chlamydial pneumonitis.

## 1. Case

**Figure 1:**
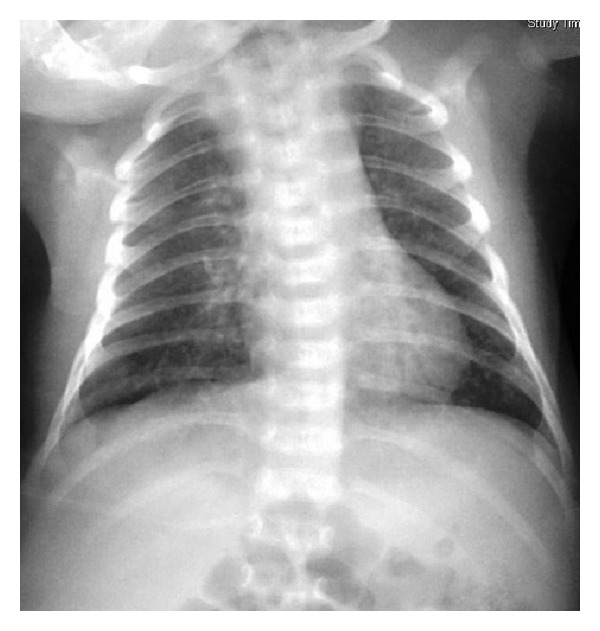
Diffuse patchiness on chest radiography in a neonate with *Chlamydia trachomatis*.

A 10-day-old full-term female presented with mild left eye discharge, which was treated with topical chlortetracycline and levofloxacin eyedrops. An eye swab yielded no bacterial pathogen and immunofluorescence test for *Chlamydia trachomatis * was equivocal. Ten days later, she presented with a cough for 5 days. There had been no fever and examination showed a well-appearing baby with no respiratory distress. Her respiratory rate was 40–48/min, chest was clear, and SaO_2_ was 100% in room air. A chest radiograph, however, revealed interstitial pneumonitis (Figure 1). Blood culture, serial complete blood counts, and C-reactive protein levels were unremarkable. Capillary blood gas showed pH 7.30, pCO_2_ 5.1 kPa, and pO_2_ 7.6 kPa. Shell vial culture of the eye swab subsequently yielded *Chlamydia trachomatis*. The child was treated with a course of antibiotics including a macrolide, and her cough resolved. Using the capillary blood gas data, assuming that PaO_2_ is not lower than the capillary PO_2_, common respiratory equations were calculated (Table 1).

## 2. Discussion

The literature was searched to address clinical questions pertinent to this case. Differential diagnoses for coughing in a neonate could include viral infections such as RSV, bacterial infection such as pertussis, gastroesophageal reflux, airway abnormalities such as tracheoesophageal fistula, and cystic fibrosis [1–3]. Isolated cough without upper respiratory tract symptoms in the family makes common respiratory viral infections unlikely. It is important to realize that not all neonates with respiratory infections present with cough, as some may become apneic instead [4]. Our case suggests that a neonate can present with cough alone without any other respiratory symptomatology. The respiratory equations confirm why her respiratory symptomatology was mild because she had relatively normal respiratory mechanics with no ARDS (acute respiratory distress syndrome), overt ventilation, oxygenation, or perfusion impairments despite abnormal chest radiography. ARDS is characterized by increased pulmonary capillary permeability and pulmonary edema that results in hypoxemia, decreased lung compliance, and bilateral diffuse alveolar infiltrates on chest radiography [5, 6]. PaO_2_/FiO_2_ indicates that her acute respiratory symptom and diffuse radiographic patchiness did not achieve ARDS severity [5–8]. Abnormal alveolar-arterial gradient signifies a significant diffusion/shunting abnormality at the alveolar-arterial interface secondary to capillary leak and relative surfactant depletion [7, 8]. Oxygenation index (OI, defined as mean airway pressure (cm) × FiO_2_ (%)/PaO_2_ (mmHg)) indirectly denotes the risk and benefit ratio for management [7]. Mean airway pressure in a spontaneous breathing neonate is assumed to be 10 cmH_2_O. These respiratory equations indicate that the patient is only mildly affected despite abnormal chest radiography. Intrapulmonary shunting (*Q*_*s*_/*Q*_*t*_) assesses ventilation-perfusion mismatch [7, 8]. In room air and with assumption that mixed venous oxygen saturation was 75%, intrapulmonary shunting was estimated to be normal in this neonate.

The literature review further suggests that it is necessary to treat neonatal chlamydial pneumonitis despite the mild clinical manifestations [9–13]. For the treatment of chlamydial ophthalmia or pneumonia, oral erythromycin for 2 weeks is recommended; additional topical therapy is unnecessary. However, in approximately 20%–30% of infants, therapy will not eradicate the organism and the infant may require a repeat oral course of antibiotics [13]. Neonatal chlamydial pneumonitis is a creepy diagnosis. Classically, chest radiography appearance may be much worse than the neonate's clinical appearance. In an otherwise afebrile well infant, treatment with a full course of macrolide antibiotic is still indicated.

## Figures and Tables

**Table 1:** Respiratory indices.

Parameters	Data	Remarks
Ventilation index	N/A	Prognostic marker of lung injury (http://www-users.med.cornell.edu/~spon/picu/calc/ventindx.htm)
Alveolar-arterial oxygen gradient	<50.9	Impaired diffusion or shunting (http://www-users.med.cornell.edu/~spon/picu/calc/aagrad.htm)
Oxygenation index	3.7	Denotes risk of treatment (http://www-users.med.cornell.edu/~spon/picu/calc/oxyindex.htm)
PaO_2_/FiO_2_ ratio	>271	Severity of lung injury: ALI < 300, ARDS < 200 (http://easycalculation.com/medical/ALI.php)
*Q*_*s*_/*Q*_*t*_	<4%	Intrapulmonary shunt and V/Q mismatch, normally <5% (http://www.medfixation.com/classic-shunt-equation-qsqt-calculation/)

*Q*_*s*_/*Q*_*t*_ = (CcO_2_ − CaO_2_)/(CcO_2_ − CvO_2_). The oxygen content of mixed arterial blood (CaO_2_) is determined by the content of oxygen in the blood that reached ventilated alveoli (CcO_2_), the content of oxygen in blood that bypassed ventilated alveoli (CvO_2_), and the proportion of the two. CcO_2_ is the content of oxygen in pulmonary capillary blood and is estimated by plugging in 100% as the saturation since the PcO_2_ (pulmonary capillary PO_2_) can be assumed to be high enough to assure 100% saturation.

Mean airway pressure in a spontaneous breathing neonate is assumed to be 10 cmH_2_O.
